# Distinct leaflet-annular remodeling pattern in severe atrial functional mitral regurgitation: a three-dimensional echocardiography study

**DOI:** 10.1186/s43044-024-00509-y

**Published:** 2024-06-24

**Authors:** Hoda Abdelgawad, Bassant Mowafy, Kawkab Khidr, Eman Elsharkawy

**Affiliations:** 1 Cardiology Department, Faculty of Medicine, Alexandria University Hospital, Champollion Street, Khartoom Square, Qism Bab Sharqi, Alexandria, Egypt; 2 King’s College Hospital NHS Foundation, Denmark Hill, London, SE5 9RS UK

## Abstract

**Background:**

Atrial functional mitral regurgitation (AFMR) is best described with normal left ventricular size and function, structurally normal mitral leaflets and dilated left atrium. Unlike the ventricular functional phenotype, changes in the annular geometry more than the tethering forces are the main culprit for mitral regurgitation. The aim of this study is to illuminate the leaflet-annular remodeling in patients with mitral regurgitation and atrial fibrillation (AF) using three-dimensional transesophageal echocardiography (3D TOE).

**Results:**

Consecutive fifty patients with AFMR underwent transthoracic echocardiography and 3D TOE: 25 patients with AF and non-mild MR and 25 patients with AF and mild MR were studied. A special mitral valve analysis software was used to accurately assess the three unique pillars for MR: annular size, leaflets’ geometry and tenting parameters.

Compared to the mild MR group, non-mild MR group had long-standing AF of more than 1 year and larger left atrial volumes (51.83 ± 12.07 ml/m^2^ vs 33.68 ± 10.97 ml/m^2^, *p* < 0.001). No significant differences were noted in respect of tenting height, area and volume (13.06 ± 2.57 mm vs 11.43 ± 2.89 mm, *p* = 0.064, 3.58 ± 1.26 cm^2^ vs 2.80 ± 0.95 cm^2^, 0.081 and 6.70 ± 2.96 cm^3^ vs 5.04 ± 2.32 cm^3^, *p* = 0.081). Conversely, the non-mild MR group had larger annular area and perimeter (16.20 ± 3.90 cm^2^ vs 13.51 ± 3.85 cm^2^, *p* = 0.023 and 14.73 ± 1.72 cm vs 13.46 ± 1.79 cm, *p* = 0.033). Similarly, the non-mild MR group had larger anterior and posterior leaflets’ areas (10.18 ± 4.02 cm^2^ vs 8.71 ± 3.08 cm^2^, *p* = 0.04 and 8.96 ± 2.60 cm^2^ vs 7.30 ± 2.17 cm^2^, *p* = 0.029). Correspondingly, more disproportionate leaflet-annular remodeling, as assessed by the ratio of total leaflets’ area to the annular area, was noted in the non-mild MR as opposed to the mild MR group (1.22 ± 0.04 vs 1.26 ± 0.04, *p* = 0.008).

**Conclusions:**

Recently, AFMR has been recognized as a remarkable entity of secondary MR with unique mechanisms. Annular dilatation with disproportionate leaflet remodeling can validate the central regurgitation. However, the call for more parameters is being emphasized to characterize the suitable candidates for percutaneous interventions.

**Graphical abstract:**

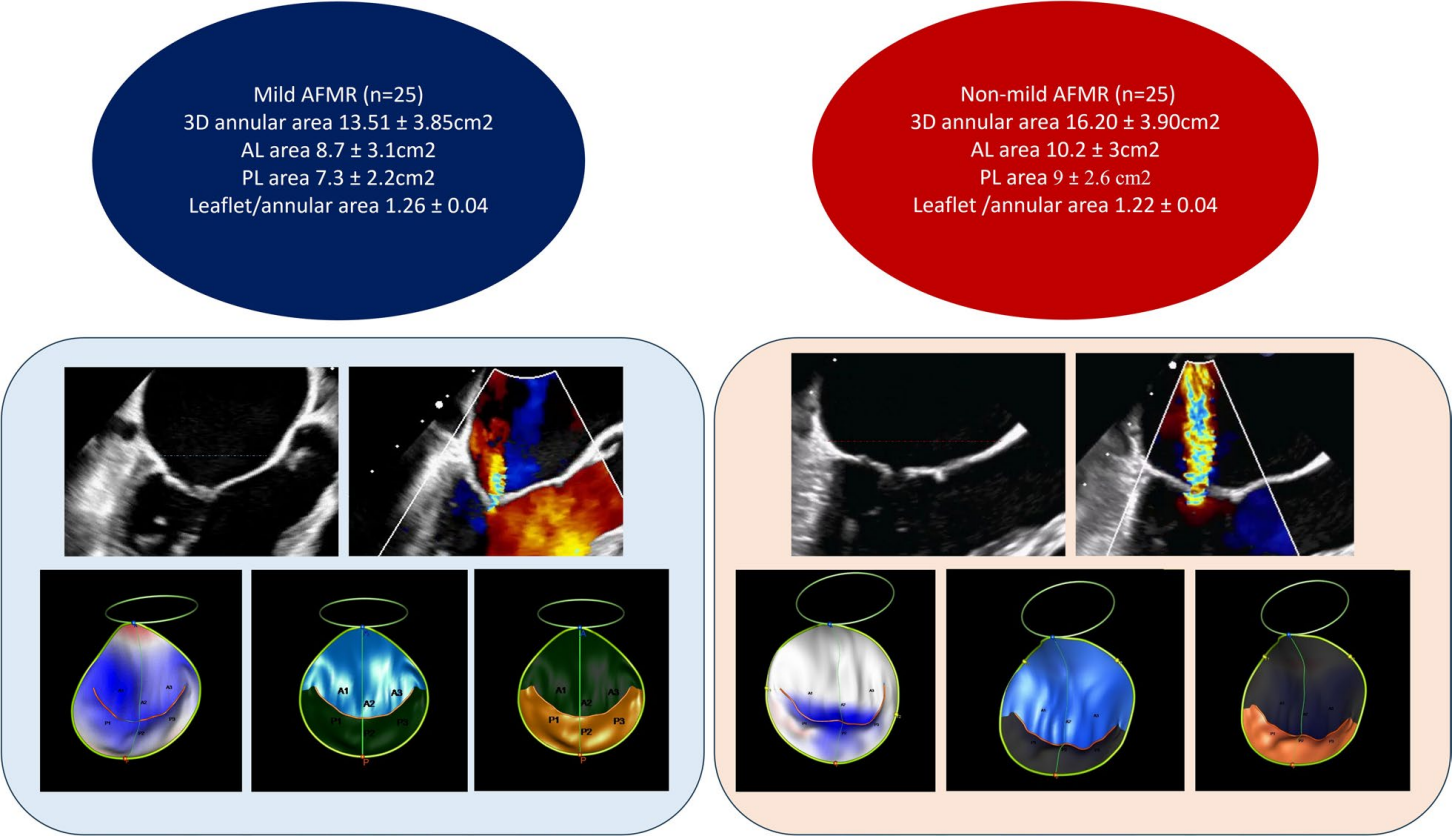

## Background

Functional mitral regurgitation (FMR) is described with geometric remodeling of the mitral valve apparatus with normal leaflet structure [1]. FMR is typically classified as an atrial and ventricular phenotype with special characteristics. The common type is the ventricular type which is associated with left ventricular remodeling in the context of ischemic heart disease or dilated cardiomyopathy [2]. On the other side, the atrial type is nominated with normal LV size, mitral leaflets and dilated left atrium. Atrial functional mitral regurgitation (AFMR) is most seen with atrial arrhythmia predominantly atrial fibrillation [3].

The traditional dilemma in the secondary type is tethering where the coaptation point is displaced into the LV away from annular plane. Tethering could be symmetrical in global LV remodeling or commonly asymmetrical in ischemic pathology seen post inferior or posterior myocardial infraction [4]. In contrary, the atrial functional phenotype had normal or minimal tethering with more annular geometrical remodeling where the coaptation point is typically found at the annular plane with central regurgitation jet and annular dilatation more than 35 mm in the anteroposterior dimeter. Like that, leaflet remodeling was expressed in patients with AFMR where compensatory growth with increase in the leaflet area and length was proposed to happen in parallel with annular dilatation till some point where the leaflet growth becomes insufficient to cover the regurgitation orifice with excessive annular dilatation [5].

This study aims to describe the leaflet-annular remodeling pattern in patients with non-mild vs mild AFMR using three-dimensional echocardiography.

## Methods

It was a single center, observational prospective cohort study at Alexandria University Main Hospital to specifically study the MR mechanism and severity in both groups, all the patients enrolled had 2D/3D Transesophageal echocardiography with further post-acquisition analysis using a special mitral valve analysis software. The institutional ethical committee approved this study.

### Study population

Consecutive fifty adult patients (≥ 18 years of age) diagnosed with atrial fibrillation and normal LV size and systolic function were studied using transthoracic echocardiography (TTE) to define the severity of the MR. Further evaluation by transoesophageal echocardiography was performed at our institution to confirm the MR severity and explore the underlying mechanism. Patients with ischemic heart disease, cardiomyopathies, any MR of primary origin and prior MV interventions were excluded.

Clinical and demographic variables including age, sex, body surface area (BSA) and AF duration were collected at the time of the TOE study.

### Echocardiographic parameters

Standard TTE and TOE examinations were performed and reported by experienced senior imaging cardiologists according to the European association of Cardiovascular Imaging guidelines utilizing Philips (EPIQ CVx version, Philips Healthcare, Andover, MA, USA) equipped with X5-1 and X8-2 phased array transducer.

The LV size including the LV dimensions using M-mode echocardiography was assessed. The LV volumes and function were evaluated using the biplane methods of disks (modified Simpson’s rule) with normal function defined as an EF ≥ 54% form females and ≥ 52% for males by TTE [6]. Severity of MR was graded using a multiparametric approach including vena contracta width (VC width) and effective regurgitant orifice area (EROA) [7].

Participants with AFMR were prospectively characterized as follows:

1. Patients with AFMR with mild MR.
2. Patients with AFMR with non-mild MR.

Left atrial size including the anteroposterior diameter and left atrial volumes were measured. 3D TTE-derived left atrial study was performed by acquiring high frame rate 3DE data sets from the apical position using the EPIQ system’s HM ACQ key.

Then, LA volumes and emptying fraction were analyzed offline using Heart Model software (Philips Healthcare, Andover, MA, USA).

3D TOE-derived mitral valve geometric measurements were performed offline using 3D zoom data sets of the mitral valve with further analysis using an automated 3D Auto MV software (TOMTEC Imaging Systems GmbH, Germany) to measure the mitral annular size including the anteroposterior and commissural annular diameters, annular circumference, and 2D/3D annular areas [8, 9]. Leaflet remodeling was determined by measuring the anterior and posterior leaflets length and areas.

In addition, tenting height (distance from the coaptation point to the annular plane) and tenting area and volume (area and volume between leaflets and annular plane) were automatically measured using the AutoMV software to allow quantification of the degree of tethering of the mitral valve. Finally, the ratio of the leaflet area to the 3D annular area was utilized as a measure of the degree of the proportional leaflet remodeling to the annular dilatation.

### Statistical analysis

Continuous data is presented as mean ± standard deviation (SD) with differences assessed using Mann–Whitney-U test. Categorical data is presented as counts and percentages, with differences evaluated using the chi-square test. Statistical analysis was conducted with SPSS 29.0 (SPSS Inc., Chicago, IL, USA) and *p* < 0.05 (two-sided) was considered statistically significant.

## Results

### Demographic, clinical, and echocardiographic characteristics of AFMR group

A total of 50 patients were diagnosed with AFMR (25 patients with mild MR and 25 patients with non-mild MR) (Table 1).

**Table 1:** Demographic characteristics of the AFMR subgroups

	Non-mild MR *n* = 25 (50%)	Mild MR *n* = 25 (50%)	*P*
Males	12 (47)	8 (33)	NS
Age (years)	50 ± 11	44 ± 10	NS
BSA (m^2^)	1.7	1.9	0.026
AF duration > 1 year	20 (80)	12 (47)	0.058

Data are *n* (%), median (IQR) or mean ± SD unless otherwise specified

*AFMR* Atrial FMR; *BMI* Body mass index; *FMR* Functional mitral regurgitation; *NS* Not significant

The non-mild MR group were 47% males with mean age of 50 ± 11 years while the mild MR group were 33% males with mean age of 44 ± 10 years. The presence of long-standing atrial fibrillation of more than 1 year was more noted in the non-mild MR (80% vs 47%, *p* = 0.05%).

### Ventricular size and function

**Fig. 1:**
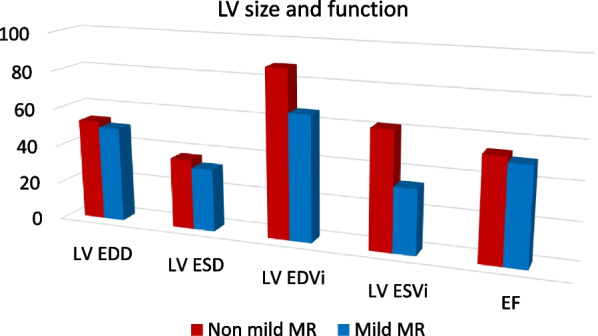
Comparison of the left ventricular size and function between studied groups

The patients showed on average LV dimensions with no between-group differences. However, the mean end-diastolic and end-systolic LV volumes in the non-mild MR group were 88 ± 25 and 62 ± 33 ml/m^2^ consecutively while the mild MR group had smaller end-diastolic and end-systolic volumes 66 ± 19 and 34 ± 11ml/m2 consecutively with *p* 0.011 and < 0.001 (Table 2, Fig. 1).

**Table 2:** Baseline LV size and function

	Non-mild MR *n* = 25 (50%)	Mild MR *n* = 25 (50%)	*P*
LV EDD (mm)	53 ± 8	50 ± 7	0.332
LVESD (mm)	37 ± 9	33 ± 6	0.263
LV EDVI (ml/m^2^)	88 ± 25	66 ± 19	0.011
LV ESVI (ml/m^2^)	62 ± 33	34 ± 11	< 0.001
EF (%)	54 ± 16	51 ± 8	0.624

*t*: Student t test, U: Mann Whitney test, *p*: *p* value for comparing between group I and II

*Statistically significant at *p* ≤ 0.05

The LV EF on average at the lower limit of normal in both studies group but no considerable difference was found: 54 ± 16% and 51 ± 8 (*p* 0.624).

### Left atrial size and function

**Fig. 2:**
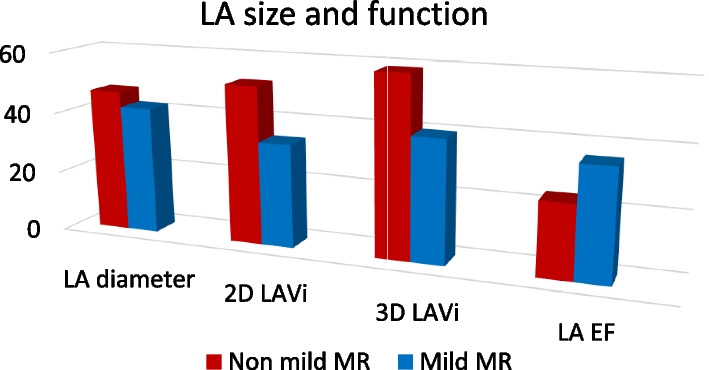
Comparison of the left atrial size and function between both groups

In both groups, 3D derived LA volumes were severely increased (Indexed LAvol > 48 ml/m^2^) in 64% of the included patients. The non-mild MR group showed more significant atrial structural remodeling than the mild MR group where the non-mild MR patients showed larger left atrial volume of 70 ± 12 ml/m^2^ in comparison to 39.8 ± 8.7 ml/m^2^ in the mild MR group. Emptying fraction of the left atrium was used as a parameter of functional atrial remodeling as the contractile function, assessed by 3D derived emptying fraction, was much lower in the non-mild MR patients as compared to the mild MR patients (23.7 ± 7.5% and 36.1 ± 19.56%, *p* 0.034). (Table 3, Fig. 2).

**Table 3:** Echocardiographic parameters of the LA size and function

LA parameters	Non-mild MR	Mild MR	*p*
LA anteroposterior diameter (mm)	46.5 ± 6.1	41.5 ± 4.9	0.02
2D LAvol (ml/m^2^)	51.8 ± 12.1	33.7 ± 11	< 0.001
3D LAvol (ml/m^2^)	58.7 ± 12	39.8 ± 8.7	< 0.001
3D EF (%)	23.7 ± 7.5	36.1 ± 19.6	0.034

*t*: Student t test, U: Mann Whitney test, *p*: *p* value for comparing between group I and II

*Statistically significant at *p* ≤ 0.05

### Mitral structural remodeling

#### Annular size

**Fig. 3:**
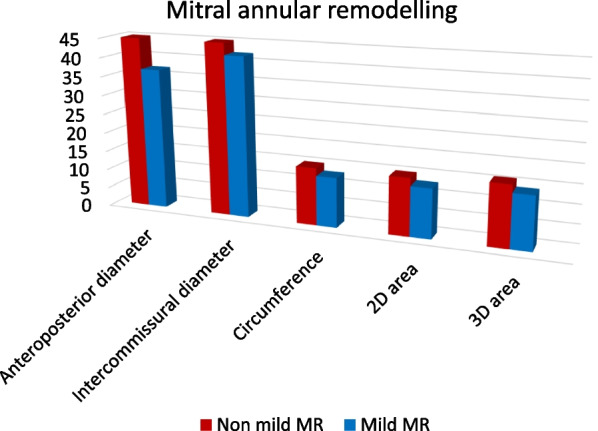
Comparison of the mitral annular size between both studied groups

Excessive annular dilatation in the anteroposterior and bi-commissural trajectories was highlighted in the non-mild MR patients in contrast to the mild MR patients. In addition, annular circumference and area were markedly increased in the non-mild MR patients against the mild MR patients (Table 4, Fig. 3).

**Table 4:** Echocardiographic parameters of mitral annular size

Annular parameters	Non-mild MR	Mild MR	*p*
Anteroposterior diameter (mm)	44.5 ± 6.7	36.7 ± 10.3	0.015
Inter-commissural diameter (mm)	44.93 ± 4.88	41.77 ± 5.83	0.026
Circumference (cm)	14.73 ± 1.72	13.46 ± 1.79	0.033
2D area (cm^2^)	15.49 ± 3.77	12.87 ± 3.71	0.023
3D area (cm^2^)	16.20 ± 3.90	13.51 ± 3.85	0.023

U: Mann Whitney test, *p*: p value for comparing between group I and II,

*SD* Standard deviation

### Leaflet area

**Fig. 4:**
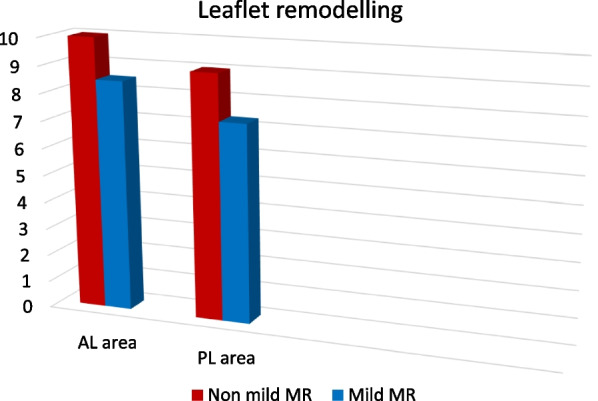
Comparison of leaflet areas between both groups

Leaflet adaptation in response to annular dilatation was recognized in the anterior and posterior leaflets where the non-mild MR group had a larger leaflet area versus the mild MR group (Table 5, Fig. 4).

**Table 5:** Echocardiographic derived leaflet area

Leaflets parameters	Non-mild MR	Mild MR	*p*
Anterior leaflet area (cm^2^)	10.2 ± 3	8.7 ± 3.1	0.04
Posterior leaflet area (cm^2^)	9 ± 2.6	7.3 ± 2.2	0.029

U: Mann Whitney test, *p*: *p* value for comparing between group I and II, *SD* Standard deviation

### Tethering forces

**Fig. 5:**
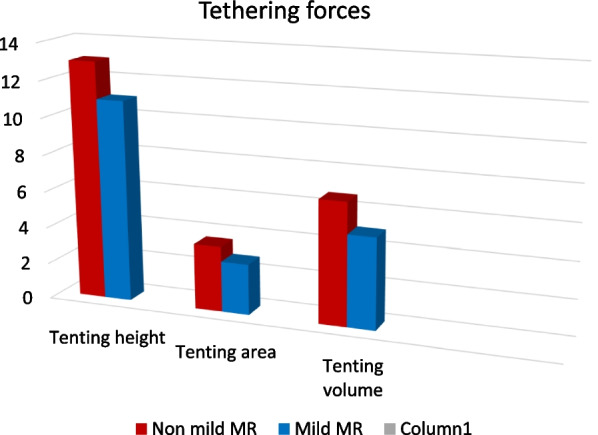
Comparison of tethering forces between both groups

Tenting parameters were utilized to assess tethering forces as a mechanism of mitral regurgitation. No remarkable difference was found between both groups in regard to tenting height, area and volume (Table 6, Fig. 5).

**Table 6:** Echocardiographic parameters of leaflet tenting

Leaflets parameters	Non-mild MR	Mild MR	P
Tenting height (mm)	13.1 ± 2.6	11.4 ± 2.9	0.064
Tenting area (mm^2^)	3.6 ± 1.3	2.8 ± 0.9	0.081
Tenting volume (ml)	6.7 ± 2.9	5.0 ± 2.3	0.081

*t*: Student *t* test, U: Mann Whitney test, *p*: *p* value for comparing between group I and II

*Statistically significant at *p* ≤ 0.05

### Leaflet-annular adaptation

Annular dilatation is commonly associated with compensatory increase in the leaflet area, but this enlargement becomes insufficient at bigger annuli causing MR by mal-coaptation of the leaflets.

**Fig. 6:**
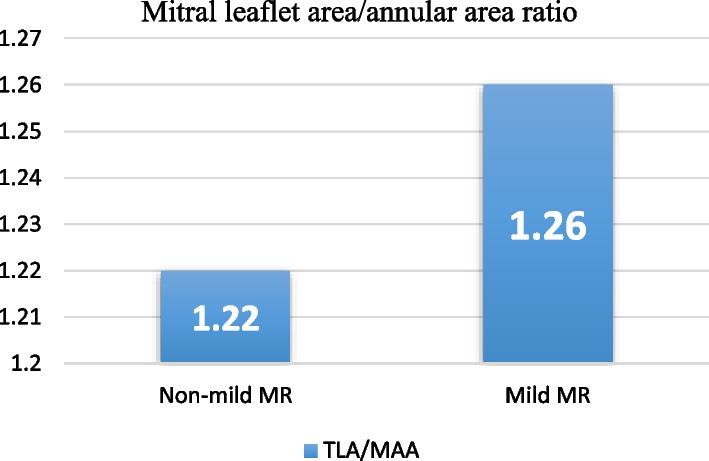
Comparison of mitral leaflet area/annular ratio between non-mild MR and mild MR patients

This was evidenced by the comparing the ratio of mitral leaflet area to annular area in both groups where it was less proportionate in the non-mild MR group. (1.22 ± 0.04 vs 1.26 ± 0.04, *p* 0.008) (Fig. 6).

## Discussion

Normal LV cavity size and systolic function in addition to concomitant mitral annular and left atrial dilatation are the main characteristics to define isolated atrial functional mitral regurgitation. However, the LV may dilate in chronic long standing AFMR. Central MR jet is typical for AFMR. Eccentric jets could be described if ventricular mechanism co-exists or if there is associated tethered posterior leaflet creating Coanda-like effect that pulls the central jet to adhere to the LA posterior wall mimicking eccentric-like jet [10].

Marked LA dilation secondary to AF causes separation of the two leaflets apart forming a coaptation gap in the context of annular dilatation. According to Carpentier, AFMR will be classified as type I MR with normal leaflet motion with annular dilatation and no or minimal tethering [11].

Assessment of MR severity should be done using the European association of cardiovascular imaging recommendations for echocardiographic assessment of native valvular regurgitation. An integrative approach to include color Doppler parameters and careful quantitative measurements of EROA and regurgitant volume, as well as qualitative supportive signs such as density, profile, and duration of the MR jet on continuous wave Doppler, pulmonary vein flow pattern, and mitral inflow E-wave velocity is a must to achieve accurate diagnosis and overcome the limitations of each parameter. The timing for echocardiographic assessment according to the AF duration is crucial. Non-significant MR is commonly seen in acute MR with rapid ventricular response that improves with restoration of sinus rhythm [12].

The assessment of MR severity is affected by AF, particularly in fast or very irregular rhythms. It is best to measure MR severity in sinus rhythm or in AF when the ventricular rate is well controlled with minimal variation in R-R intervals. It is recommended to use the indexed beat method by selecting a beat for which the preceding and following R-R intervals are similar [13].

Multiple mechanisms of the AFMR have been proposed however, the most common proposals are: (1) mitral annular dilatation in parallel to insufficient leaflets remodeling causing leaflet malcoaptation [14] and (2) leaflets tethering where the LA enlargement displaces the posterior mitral annulus onto the crest of the LV inlet causing tethering of the posterior leaflet by increasing the annulopapillary muscle distance. In addition, the displacement of the posterior mitral annulus may cause a counterclockwise torque of the anterior mitral annulus increasing the tethering of the papillary muscles and causing tenting of the anterior mitral leaflet [15, 16].

We have found that atrial dilatation and dysfunction were more noted in the AFMR with non-mild MR than the mild MR group suggesting an atriogenic origin of the MR in the context of AF. Considering these findings, it was found that LA reservoir function but not LA size is a robust predictor of outcome in significant AFMR. This provides mechanistic insights into the interplay of functional versus geometric LA changes in AFMR [17].

Accordingly, we aimed to have a better understanding of the underlying mechanisms of AFMR in mild and non-mild regurgitation. First, non-mild MR was more frequently seen in chronic AF for more than a year. We have studied both hypotheses in mild and non-mild AFMR. Leaflet tenting was noted in both groups of AFMR however, no significant difference was found between both groups.

In respect of annular remodeling, increased annular dimensions were measured in the non-mild AFMR in comparison to the mild AFMR group. In addition, the anterior and posterior leaflets areas were markedly increased in non-mild AFMR. Interestingly, in patients with non-mild MR, the ratio of mitral leaflet to annular area (representing the mitral annular surface effectively covered by the leaflets) was significantly reduced as compared with patients with mild MR (1.22 ± 0.04 versus 1.26 ± 0.04, respectively; *P* = 0.008) suggesting that insufficient mitral leaflet remodeling to compensate the mitral annulus dilatation may be pivotal in the development of significant AFMR.

In agreement with our results, the geometry of the mitral valve assessed with 3D Transesophageal echocardiography of 28 patients with AF and significant MR was compared with that of 56 AF patients without MR and 16 normal controls matched by age and sex. LA dimensions, mitral annulus size, and anterior and posterior mitral leaflets were significantly larger in patients with AF and MR as compared with the other 2 groups. However, patients with MR showed significantly smaller total leaflet area relative to the mitral annulus area compared with AF patients without MR and controls (1.29 ± 0.10 versus 1.65 ± 0.24 versus 1.70 ± 0.29, respectively; *P* < 0.001). Each 1% decrease in the total leaflet area to mitral annulus area ratio was independently associated with significant MR (odds ratio 0.76, 95% confidence interval 0.65–0.89; *P* < 0.001) [11, 14, 18].

In concordance to these findings, recent studies using three-dimensional echocardiography, have shown that significant functional MR can sometimes occur in AF patients with significant dilatation of mitral annulus and left atrium. Additional contributors such as atriogenic leaflet tethering, annulus area to leaflet area imbalance resulting from insufficient leaflet remodeling and reduced annular contractility, increased valve stress by flattened saddle shape of the annulus and left atrial dysfunction may be important triggers of atrial functional MR in the presence of dilated mitral annulus and left atrium [19].

Earlier studies were supportive of the atriogenic tethering theory of the leaflets as the only mechanism of MR in AF that causes annular displacement and leaflets’ tenting [15] in contrary to our study which showed no significant difference in the tenting parameters between significant and non-significant MR groups.

Therefore, disproportionate leaflet-annular remodeling is the cornerstone mechanism in atrial functional MR. Whether these changes will revert after effective restoration of sinus rhythm remains to be investigated. Probably, patients with AF and significant MR because of annular dilatation and insufficient leaflet remodeling may show more diseased LA with low probability of recovering sinus rhythm.

### Limitations

The present study is a single-center study. Our patient cohort may not be representative for all patients with atrial fibrillation due to differences in population genetics and epidemiology of AF and would ideally be validated in an external patient cohort.

## Conclusion

Functional mitral regurgitation secondary to atrial remodeling is the rising star in patients referred for mitral valve interventions. Many researchers have described the main determinants and pathophysiological factors of the occurrence and progression of AFMR. Insufficient leaflet remodeling to compensate for the annular atrial dilatation is the main culprit for AFMR progression and to lesser extent the imbalance between the tethering and closure forces because of LV remodeling may contribute to initial formation of AFMR. Whether early restoration of atrial sinus rhythm could help prevent or delay the MR occurrence needs to be addressed. This raises the call for more studies of novel therapeutic approaches aiming to reduce MR severity and to improve patient outcomes.

## Data Availability

The data underlying this article will be shared upon request to the corresponding authors.

## Abbreviations

2D: Two dimensional
3D: Three dimensional
AF: Atrial fibrillation
AFMR: Atrial functional mitral regurgitation
FMR: Functional mitral regurgitation
MR: Mitral regurgitation
MV: Mitral valve
EDD: End diastolic diameter
EDVI: Indexed end diastolic volume
EROA: Effective regurgitant orifice area
ESD: End systolic diameter
ESVI: Indexed end systolic volume
LA: Left atrium
LA EF: Left atrial emptying fraction
LAVmax: Maximum left atrial volume
LAVmin: Minimum left atrial volume
LV: Left ventricle
TOE: Transoesophageal echocardiography
TTE: Transthoracic echocardiography
